# Innate lymphoid cells in bone marrow and peripheral blood of healthy individuals and in bone marrow of patients with myelodysplastic syndromes

**DOI:** 10.3389/fimmu.2025.1568875

**Published:** 2025-06-11

**Authors:** Laiz C. Bento, Guilherme Martelossi Cebinelli, Nydia Strachman Bacal, Luciana Cavalheiro Marti

**Affiliations:** ^1^ Clinical Pathology Laboratory, Hospital Israelita Albert Einstein, São Paulo, Brazil; ^2^ Experimental Research Department, Hospital Israelita Albert Einstein, São Paulo, Brazil

**Keywords:** innate lymphocytes, myelodysplastic neoplasms, flow cytometry, ILC2 - group 2 innate lymphoid cell, ILC1 -group 1 innate lymphocyte, ILC3 -group 3 innate lymphocyte

## Abstract

**Introduction:**

Innate lymphoid cells (ILCs) are a recently characterized subset of lymphocytes with critical effector and regulatory functions. Based on distinct phenotypic markers and cytokine secretion profiles, ILCs are classified into three subtypes: ILC1, ILC2, and ILC3. The bone marrow (BM) microenvironment in myelodysplastic neoplasms (MDS) is characterized by heightened cytokine levels and dysregulated inflammatory responses, potentially affecting immune cell composition. In this study, we aimed to assess the frequency and absolute number of ILCs in the BM and peripheral blood (PB) of healthy individuals (HI) and compare them to those in MDS-BM.

**Methods:**

ILC subsets were identified and quantified using multiparameter flow cytometry and histological analysis of BM and PB samples.

**Results:**

ILC1 populations were consistently detected in all PB and BM samples. In contrast, ILC2 and ILC3 subsets were present in both BM and PB of healthy individuals but were largely absent in MDS-BM samples. Additionally, our findings suggest the existence of a potential maturation trajectory for BM-resident ILCs.

**Discussion:**

These data indicate a disruption in the ILC maturation continuum within the MDS-affected BM compared to healthy BM, potentially contributing to the immunopathogenesis of MDS.

## Introduction

In the last 10 years, a new family of lymphocytes has been described as innate lymphoid cells (ILC) (1–7). Innate lymphocytes are characterized by lack of expression of T cell receptor and play significant roles in innate immunity, as effectors and regulators (8, 9). These cells have been classified into three main groups based on their phenotypic characteristics, cytokine profile and transcription factors required for their differentiation, as follows: type 1 innate lymphocytes (ILC1), type 2 innate lymphocytes (ILC2) and innate lymphocytes type 3 (ILC3) (10).

ILC1 are cells that express T-bet, produce interferon γ (IFN-γ) and act in the defense against intracellular paathogens (10). Currently, ILC1 can be divided in two subsets based on their expression profiles. One subset of ILC1 is described to express CD127^low^, CD103, NKp44, CD94 and CD56. They are responsive to interleukin (IL) 12 and IL-15, but their relationship with NK cells has not yet been clearly described in the literature. The other subset is described to express CD127^high^, CCR6 and CD161, and does not express NKp44, CD94, or CD56. This subset is predominantly found in the lamina propria (1, 5, 7).

ILC2 express GATA3 and are cells that produce IL-4, IL-5 and IL-13 in response to stimulation with IL-25 and IL-33. They also respond in the presence of thymic stromal lymphopoietin (TSLP) (11). These cytokines play an important role in the innate immune response against helminths, and nematodes, and contribute to allergies. This subset expresses CD7, CD127, CD161, CCR6 and CRTH2. In humans, ILC2 have been described in the intestine, skin, peripheral blood, and lung (1, 6, 12).

ILC3 are described in the tonsils, in the lamina propria of the human intestine, in the spleen and lymph nodes (10, 13). This subset is known to express the receptor retinoic acid γt (RORγt) (7, 13). Furthermore, these cells express CD7, CD127, CD161 and NKp46, but they do not express CRTH2 (6). Depending on the stimulus, ILC3 can produce tumor necrosis factor (TNF), IL-17 and granulocyte macrophage colony-stimulating factor (GM-CSF), thereby promoting antibacterial and anti-inflammatory responses or participating in tissue repair (1, 7).

ILCs have been more thoroughly characterized recently and have emerged as an effector and regulatory family of innate immune responses that participate in various processes such as pathogen resistance, inflammation, and the maintenance of homeostasis (14). Defects in both innate and adaptive immune response have been implicated in the pathogenesis of Myelodysplastic Neoplasms (MDN) (15). Chronic inflammatory responses and unresolved persistent infections are correlated with increased susceptibility to cancer, a relationship that has been recognized over the years. The literature highlights an essential role for ILC in chronic inflammatory responses, suggesting a potential involvement of these cells in the development of pathologies including cancer (13, 16, 17).

The immune response plays a crucial role as a barrier to cancer, with the immune system being known for its ability to eliminate tumor cells. However, the role of innate lymphocytes in this process remains poorly understood. Human ILC are lymphocytes subsets that have not been well defined in the literature, and their immunophenotypic patterns are poorly described in the peripheral blood of healthy individuals. Furthermore, to date, they have not been described in healthy human bone marrow.

In this study, we identified, characterized, and quantified the three populations of innate lymphocytes in the bone marrow and peripheral blood of healthy individuals. Additionally, we have compared the typical profile of innate lymphocytes with that found in bone marrow from patients with Myelodysplastic Neoplasms (MDN), using a maturation curve developed by us and based on existing literature. Moreover, we compared the morphological characteristics of the three innate lymphocytes with those of T, B and NK cells.

## Materials and methods

### Samples

This study included 40 bone marrow (BM) samples from patients undergoing orthopedic surgery, all of whom had hemogram results within normal ranges, with a mean age of 37 years old (range 20 to 86). We also included 39 peripheral blood (PB) samples from healthy individuals with a mean age of 38 years old (range 20 to 65) as demonstrated by **Table 1**. All samples were from healthy individuals over 18 years old, without clinical follow-up due to hematological disease and without treatment with growth factors, chemotherapy, cytotoxic or immunosuppressive drugs, they also referred no other comorbidities. Additionally, we included 25 bone marrow samples from patients diagnosed with Myelodysplastic Neoplasia (MDN), with a mean age of 75 years old (range 53 to 92). The disease classification is detailed in **Supplementary Table S1**. As a limitation, no additional information on MDN patients’ treatments or comorbidities was available. All samples were collected in a tube containing the anticoagulant potassium ethylenediaminetetraacetic acid (EDTA).

**Table 1:** Demographic data for healthy samples of peripheral blood and bone marrow.

Sample	N	Age Median	Gender	TCN (mm^2^) Median	Lymphocytes (mm^2^) Median	Monocytes (mm^2^) Median	Granulocytes (mm^2^) Median	Hb Median	Ht Median
Peripheral blood	39	38 (20-63)	11(Male) 27 (Female)	7,230 (4,020-11,430)	2,620 (1,160-4,210)	570 (230-1,130)	4,120 (2,170-7,220)	41 (35.1-47.9)	13.9 (12.4-16.7)
Bone Marrow	40	36 (20-86)	26(Male) 14 (Female)	25,720 (6,650-88,760)	5,00 (2,050-13,780)	1,800 (578-6,720)	18,600 (3,310-69,190)	39.9 (30.2-47.9)	13.5 (8.9-16.8)

This study complied with the Declaration of Helsinki and was performed in accordance with the approval of the Hospital Israelita Albert Einstein Ethics Committee under the Certificate of Presentation for Ethical Appreciation (CAAE) number 82751318.2.0000.0071 and 31895320.0.3001.0068. All participants signed an informed consent form before the collection of blood or bone marrow samples.

### Characterization of innate lymphocytes by flow cytometry

Bone marrow and peripheral blood samples were processed within 48 hours of collection, and the material cellular viability was assessed by flow cytometry using the vital dye 7-Actinomycin D (7-AAD). Only samples with viable cells percentage of 90% or higher were processed and included in the study. For the characterization and analysis of innate lymphocytes, 10-color panels with the following monoclonal antibodies were used:

**Tube 1:** (*)LIN2-FITC, CRTH2-PE (clone: BM16), ICOS-PE Alexa 610 (clone: C398.4A), CD94-PerCP Cy5 (clone: DX22), NKp46-PC7 (clone: 29A1.4), NKp44-APC (clone:44.189), CD7- Alexa 700 (clone: 8H8.1), CD45-APC-Cy7 (clone: 2D1), CD127-BV421 (clone: HIL-7R-M21), and CD161-BV510 (clone: DX12);
**Tube 2:** (*)LIN2-FITC, CRTH2-PE (clone: BM16), CD38-ECD (clone: LS198-4-3),CD34 PerCP (clone: 8G12), NKp46-PC7 (clone: 29A1.4), CD117-APC (clone: 104D2), CD7-Alexa 700 (clone: 8H8.1), CD45-APC-Cy7 (clone: 2D1), CD127-BV421 (clone: HIL-7R-M21), and CD161-BV510 (clone: DX12).

Antibodies were provided by BD Biosciences and Beckman Coulter.

(*) Anti-human Lineage cocktail 2 (*) LIN2 is cocktail of the following antibodies: CD3-FITC (SK7), CD14-FITC (SJ25C1), CD19-FITC (L27), CD20-FITC (MφP9), and CD56-FITC (NCAM16.2).

The technical staining process followed a previously validated standard operating procedure, and this characterization was previously described (18). Briefly, the samples were concentrated, lysed, and labeled. After 20 minutes of incubation in the dark at room temperature, the samples were washed and resuspended with 500 µL of PBS. The samples were acquired using Navios Flow Cytometer (Beckman Coulter^®^). The median number of events acquired varied according to sample type: the events in the CD45 region for healthy bone marrow, it was approximately 8,594,165 events per tube (range 207,521 – 10,906,193), for peripheral blood, it was about 4,275,539 events per tube (range 255,692 – 4,947,840) and for MDN bone marrow, it was about 1,020,317 events per tube (range 144,997 – 9,134,999). The analysis was conducted using INFINICYT software (Cytogonos, Salamanca, Spain).

### Gating strategy

In summary, the initial analysis strategy was performed using the FSC-H (FS PEAK) versus FSC-A bi-parametric dot plot to remove cells doublets., which are cells passing simultaneously through the laser. Next, gating was performed in FSC versus SSC, were the thresholds for these parameters excluded cellular debris, platelets, and other debris. The population positive for CD7, CD45, CD127 and CD161 and negative for LIN2 was separated and divided into CRTH2 positive/NKp46 negative and CRTH2 negative/NKp46 positive (**Supplementary Figure S1**). Additionally, this same strategy was used to determine a possible maturation curve through the CD34 and CD127 positive population (**Supplementary Figure S2**).

Although CD117 (c-KIT) is commonly included in gating strategies to enhance the discrimination among ILC subsets, being highly expressed in ILC progenitors and ILC1, less so in ILC2, and absent in ILC3—we chose not to rely on this marker in our analysis. This decision was based on the context of our study, which involves comparing healthy bone marrow with bone marrow from Myelodysplastic patients, where c-KIT expression may be altered due to potential mutations (19).

To determine the positive and negative populations, instead of fluorescence minus one, we used internal reaction controls consisting of distinct immune cell populations, namely granulocytes, lymphocytes, and monocytes (**Supplementary Figure S3**). These populations were selected based on their well-characterized expression profiles for the markers of interest. Granulocytes, lymphocytes, and monocytes exhibit differential expressions of these markers, serving as natural reference points within the same sample. Their distinct fluorescence intensity distributions enabled us to establish gating strategies, ensuring accurate discrimination between positively and negatively stained populations. This approach minimizes variability and enhances the reliability of our analysis by providing internal validation within each experiment.

The cell viability of the samples was determined using 7AAD (**Supplementary Figure S4**).

Furthermore, an analysis was performed using R Studio software to generate UMAP algorithms that highlighted the main differences between precursors and ILCs in healthy and MDN bone marrow. Briefly, flow cytometry data was preprocessed using FlowJo v10.9 (BD Biosciences). The gating strategy included single cells, removed outliers, and selected and exported the Lineage2-, CD34+, and CD127+ cells into a new. fcs file, which was imported into R Studio. Next, the data were transformed using a hyperbolic arcsin function and normalized. Dimensional reduction was performed by uniform manifold approximation and projection (UMAP), and the FlowSOM algorithm was used for automatic clustering (20, 21). Heatmaps with median expression values were generated and used to identify clusters in ILC types 1, 2, 3, or CD34+ CD127+ cells.

### Flow cytometry cell sorting

ILC1, ILC2, ILC3, T, B, and NK cells were separated using cell-sorting (BD FACSAria Fusion–BD Biosciences – San Jose, CA). For ILCs the cell sorting was performed using the antibodies described in ILCs characterization by flow cytometry tube 1. For T, B, and NK cells, the antibodies used were CD56-PE (clone: C218), CD19 -PC7 (clone: J3-119), CD3 APC (clone: SK7) and CD45 KO (clone: J33).

Cell sorting after staining was conducted using a purity mask, 100µM nozzle, and a flow rate of 3 to 8 thousand events per second.

### Cell morphological analysis

The morphological analysis of ILC1, ILC2, ILC3, T, B and NK cells from peripheral blood samples, was performed after cell sorting. Cells were prepared using cytospin and stained with Rosenfeld dye (methylene blue eosin Maygrünwald combined with azurine methylene diluted in methanol). Briefly, to prepare the slide, 50 µL of each sample previously separated through cell-sorting as described earlier was used. Slides were prepared with filter paper and a plastic device, placed in a metallic structure. The sample was pipetted into the plastic device, and the slides were centrifuged at 1000 rpm for 5 minutes (Cytospin ™ 4 Citocentrifuge). After centrifugation and removal of the filter, the slides were left at room temperature for 30 minutes to dry. Next, cells were stained by adding 0.5mL of Rosenfeld dye for 2 minutes and then 1mL of phosphate buffer for 6 minutes. The stained samples were analyzed at 100x magnification on an Eclipse 80i microscope (Nikon).

### Statistical analysis

All analyses were conducted separating healthy BM and PB samples, and MDN-BM. Gender distributions were described within each groups using relative frequency, and group associations were assessed using the chi-square test. Other parameters were described across groups using minimum and maximum medians and compared between groups using Mann-Whitney tests. Statistical significance was set at a level of 5% (α=5%).

## Results

### Purity and viability of healthy bone marrow samples

To assess samples hemodilution, we employed a method described by Pont et al. (22). This method utilizes the ratio (**r**) of immature granulocytes (promyelocytes, myelocytes, metamyelocytes and rods) by neutrophils percentage to define the samples purity. The results obtained classified 29 samples (72.5%) as high purity (**r**≥1.2), 10 samples (28.6%) as moderate purity (**r** between 0.5 and 1.2), and 1 sample (2.85%) as low purity (**r**<0.5). Furthermore, cell viability was assessed using 7AAD dye in all samples, with results consistently above 90%, and a median of 96.1% (range 91.1%-99.9%).

### ILC1, ILC2 and ILC3 in healthy and MDN bone marrow and peripheral blood

**Figure 1:**
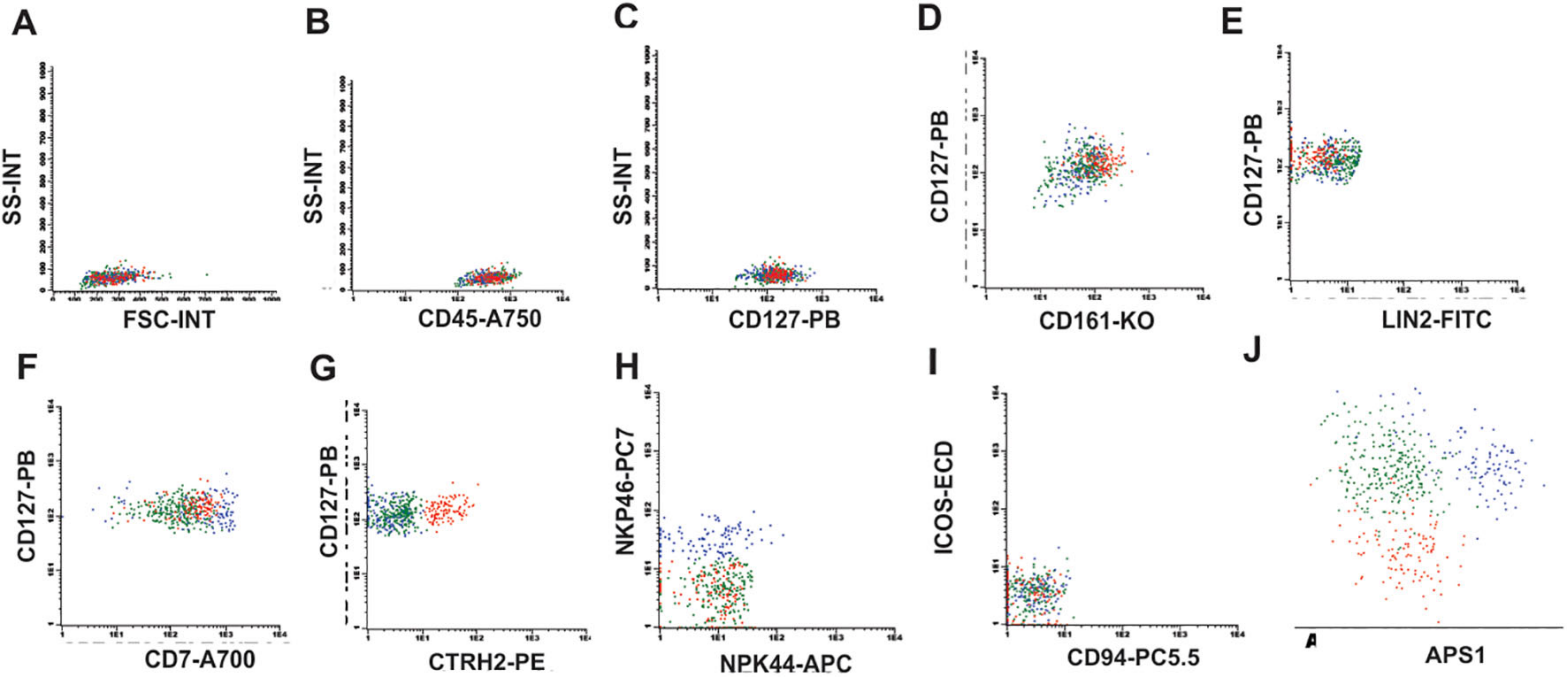
ILC identification strategy. **(A)** Flow cytometry plots showing the initial gate in FSC-A vs SSC-A, **(B)** CD45 positive cells, **(C)** CD127 positive cells, **(D)** Double positive staining for CD127 and CD161, **(E)** Negative staining for Lineage 2 (Lin2), **(F)** Positive staining for CD7 showing the population of ILC1 (green), **(G)** Positive staining for CRTH2 for ILC2 (red), **(H)** Positive staining for NKp46 identifying the ILC3 population (blue) with negative NKp44, **(I)** ICOS and CD94 negative for ILC1, 2 and 3, **(J)** APS graph showing that ILCs are distinct populations.

ILC1s were identified through the expression of CD7, CD45, CD127, CD161, absent/low NKp46 and absent LIN2, and CRTH2. ILC2 were identified through the positive expression of CD7, CD45, CD127, CD161, CRTH2, absent/low NKp46 and absent LIN2. ILC3 were identified through the positive expression of CD7, CD45, CD127, CD161 and NKp46 and absence of CRTH2 and LIN2. All analysis were demonstrated in **Figure 1** and **Supplementary Figure S1**. Additionally, ILC1, ILC2 and ILC3 frequencies determination were performed within the total lymphocyte population.

**Figure 2:**
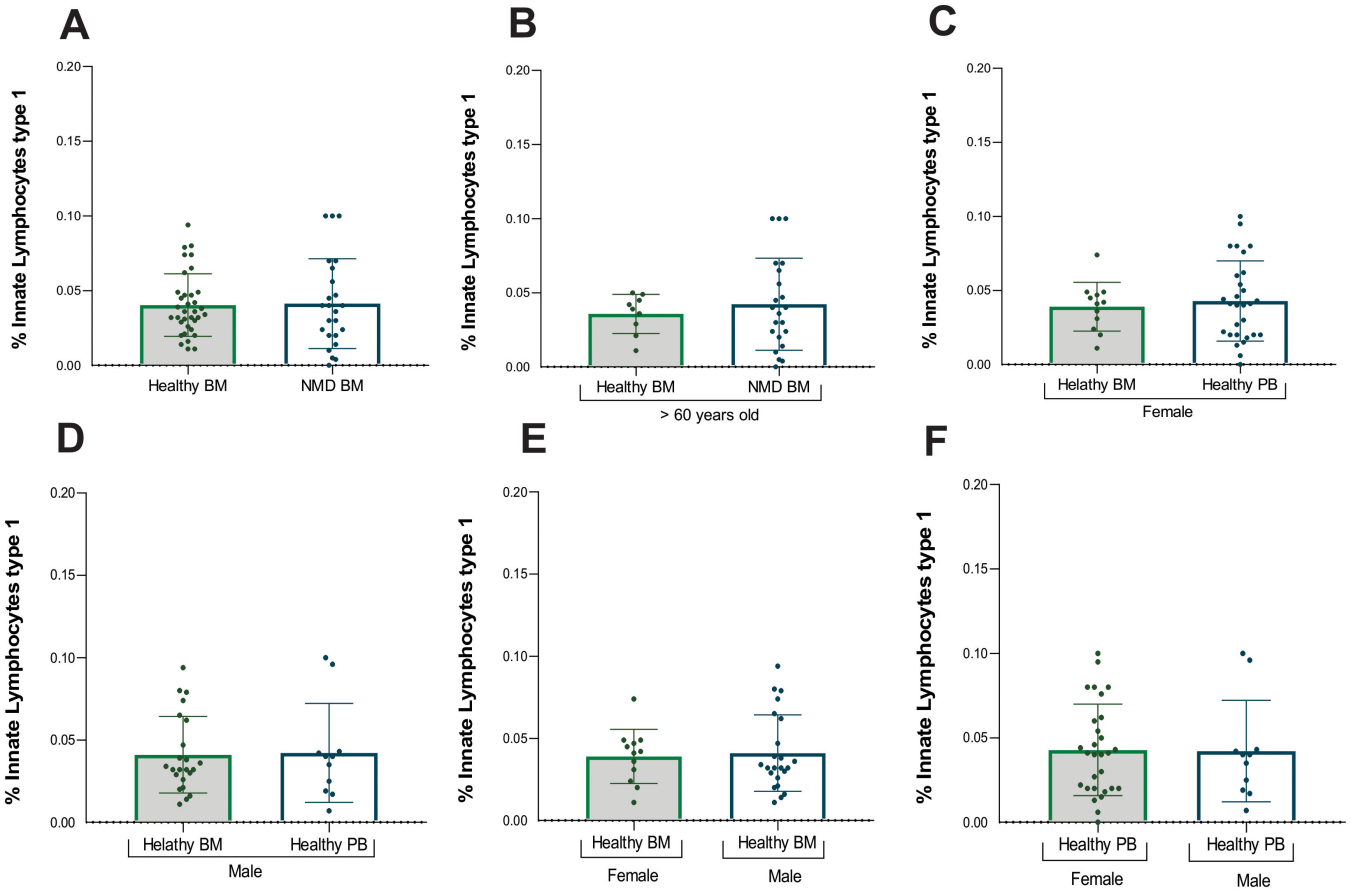
Statistical analysis of ILC1. **(A)** Analysis between samples of healthy bone marrow (n=40) and MDN marrow (n=25), **(B)** Analysis between samples of healthy bone marrow (n=9) and MDN marrow (n=25) normalized by age (> 60 years old), **(C)** Analysis between samples of healthy bone marrow (n=40) and peripheral blood (n=28) from the female population **(D)** Analysis between samples of healthy bone marrow (n=40) and peripheral blood (n=11) from the male population, **(E)** Analysis between samples of healthy bone marrow of female population (n= 14) and healthy bone marrow of male population (n=26), **(F)** Analysis between samples of healthy peripheral blood of female population (n=28) and healthy peripheral blood of male population (n=11). An outlier was identified on prism program using ROUT (Q=1%), this outlier was removed from the original data analysis, however, the presence or absence of the outlier data has not modified the significance of the comparison between groups.

No significant difference was observed between healthy and MDN-BM samples for ILC1 (**Table 2**; **Figure 2A**). In the logistic regression study, after adjusting for age, no influence of ILC1 was identified in discriminating normal MO and SMD (p = 0.867). Comparative analysis of ILC1 in BM samples from individuals older than 60 years also showed no statistical difference between healthy and MDN bone marrow (**Figure 2B**). ILC1 frequencies were further analyzed by gender, and no statistical difference was found between healthy bone marrow and peripheral blood samples for either females or males (**Supplementary Tables S2**, **S3**; **Figures 2C, D**). Moreover, there was no statistically significant difference between the genders in both bone marrow and peripheral blood samples (**Figures 2E, F**).

**Table 2:** Results ILC1frequency in healthy bone marrow, peripheral blood, and bone marrow of MDN patients.

GROUP	ILC1		
	HEALTHY BONE MARROW	PERIPHERAL BLOOD	MDN BONE MARROW
	Median	Median	Median
**ILC1 (LR) %**	0.036 (0.011 – 0.49)	0.04 (0 – 0.1)	0.04 (0 – 0.3)
**ILC1 (EVN)**	319 (77 - 735)	520 (0 - 1890)	264 (0 - 2375)
**ILC1 (LR - EVN)**	8,594,165 (207,521 – 10,906,193)	4,275,539 (255,692 – 4,947,840)	1,020,317 (144,997 – 9,134,999)

LR, Lymphocyte region; EVN, Events Number.

**Figure 3:**
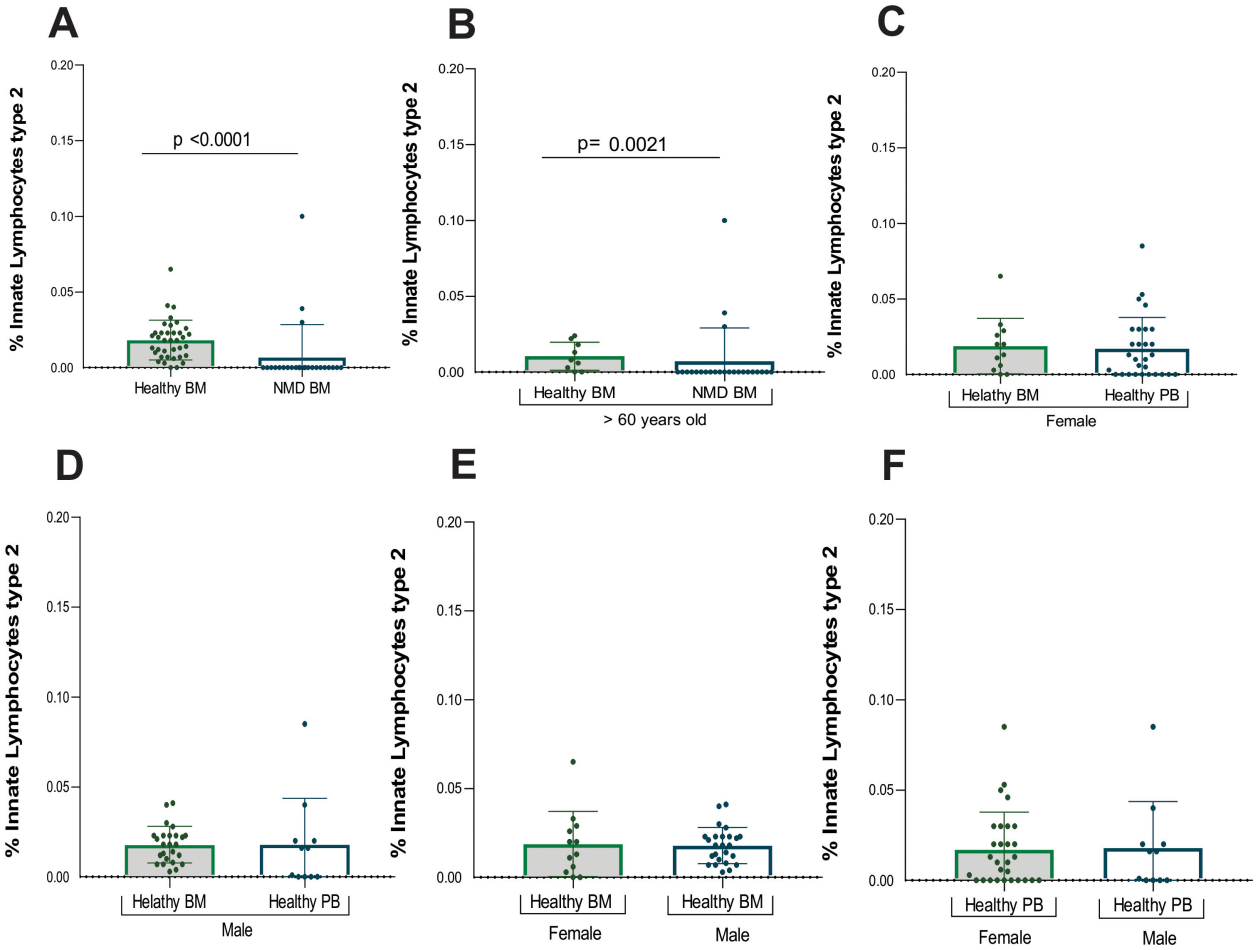
Statistical analysis of ILC2. **(A)** Analysis between samples of healthy bone marrow (n= 40) and MDN marrow (n=25), **(B)** Analysis between samples of healthy bone marrow (n= 9) and MDN marrow (n= 25) normalized by age (> 60 years old), **(C)** Analysis between samples of healthy bone marrow (n= 40) and peripheral blood (n=28) from the female population **(D)** Analysis between samples of healthy bone marrow (n=40) and peripheral blood (n= 11) from the male population, **(E)** Analysis between samples of healthy bone marrow of female population (n=14) and healthy bone marrow of male population (n=26), **(F)** Analysis between samples of healthy peripheral blood of female population (n=28) and healthy peripheral blood of male population (n=11).

Mostly bone marrow from MDN patients displayed absence of ILC2, among the bone marrow samples. There was a significant difference between healthy and MDN individuals (p<0.0001). This significant difference was also observed when analyzing only individuals older than 60 years old (p=0.0021) as shown in (**Table 3**; **Figures 3A, B**). This result indicates that the absence of ILC2 in MDN-BM is a consequence of the disease rather than the aging process.

**Table 3:** Results of ILC2 frequency in healthy bone marrow, peripheral blood and MDN bone marrow.

GROUP	ILC2		
	HEALTHY BONE MARROW	PERIPHERAL BLOOD	MDN BONE MARROW
	Median	Median	Median
**ILC2 (LR) %**	0,018 (0 - 0,065)	0,01 (0 - 0,085)	0 (0 - 0,1)
**ILC2 (EVN)**	118 (0 - 657)	141 (0 - 682)	0 (0 - 631)
**ILC2 (LR - EVN)**	8,594,165 (207,521 – 10,906,193)	4,275,539 (255,692 – 4,947,840)	1,020,317 (144,997 – 9,134,999)

LR, Lymphocyte region; EVN, Events Number.

However, three MDN patients showed a small frequency of ILC2 in bone marrow and given the relatively small sample size, the inclusion of multiple variables in a logistic regression model could reduce statistical power. Among them, two had MDN with a low number of blasts, and one had MDN with a low number of blasts along with an isolated 5q deletion.

Additionally, ILC2 frequencies were also evaluated by gender, revealing no statistical difference between healthy bone marrow and peripheral blood samples for either females or males (**Supplementary Tables S4**, **S5**; **Figures 3C, D**). Furthermore, no statistically significant difference was found between genders in both BM and PB samples (**Figures 3E, F**).

**Figure 4:**
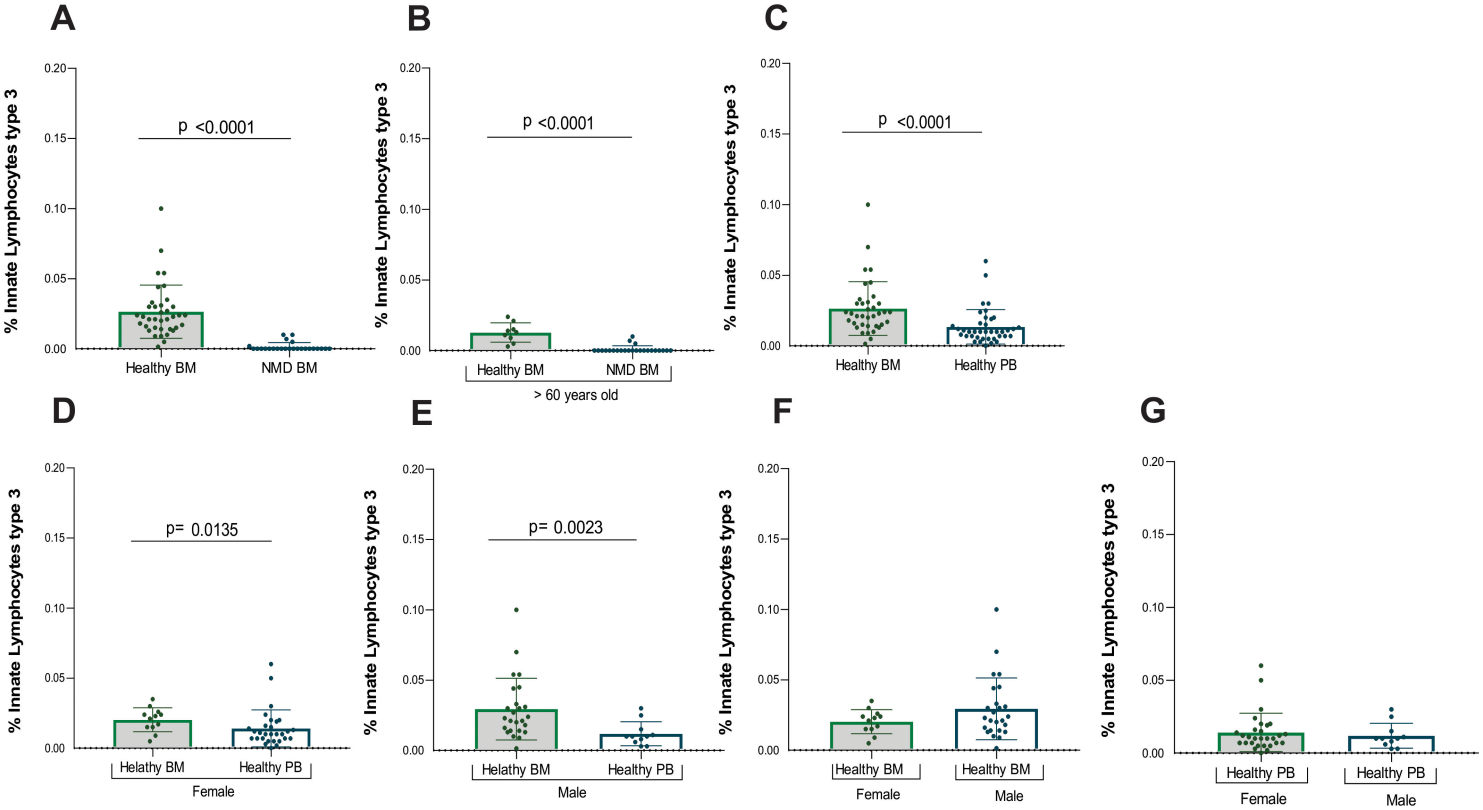
Statistical analysis of ILC3. **(A)** Analysis between samples of healthy bone marrow (n= 40) and MDN marrow (n=25), **(B)** Analysis between samples of healthy bone marrow (n=9) and MDN marrow (n= 25) normalized by age (> 60 years old), **(C)** Analysis between all healthy marrow (n= 40) and peripheral blood (n= 39), **(D)** Analysis between samples of healthy bone marrow (n= 40) and peripheral blood (n=28) from the female population **(E)** Analysis between samples of healthy bone marrow (n= 40) and peripheral blood (n= 11) from the male population, **(F)** Analysis between samples of healthy bone marrow of female population (n= 14) and healthy bone marrow of male population (n=26), **(G)** Analysis between samples of healthy peripheral blood of female population (n=28) and healthy peripheral blood of male population (n=11).

Mostly MDN-BM patients displayed an absence of ILC3. A significant difference was observed between healthy and MDN bone marrow samples (p<0.0001) (**Table 4**), and this significant difference persisted when analyzing only individuals older than 60 years old (p<0.0001) (**Figures 4A, B**). This result indicates that the absence of ILC3 in MDN bone marrow is a consequence of the disease rather than the aging process.

**Table 4:** Results of ILC3 frequency in healthy bone marrow, peripheral blood and MDN bone marrow.

GROUP	ILC3		
	HEALTHY BONE MARROW	PERIPHERAL BLOOD	MDN BONE MARROW
	Median	Median	Median
**ILC3 (LR)%**	0.023 (0.0015 - 0,1)	0.01 (0 – 0.06)	0 (0 – 0.01)
**ILC3 (EVN)**	185 (34 - 716)	137 (0 - 1101)	0 (0 - 222)
**ILC3 (LR -EVN)**	8,594,165 (207,521 – 10,906,193)	4,275,539 (255,692 – 4,947,840)	1,020,317 (144,997 – 9,134,999)

LR, Lymphocyte region; EVN, Events Number.

However, five MDN patients exhibited a small frequency of ILC3 in the bone marrow and given the relatively small sample size, the inclusion of multiple variables in a logistic regression model could reduce statistical power. Among them, one had MDN with a low number of blasts, another had MDN with a low number of blasts and an isolated 5q deletion, two had MDN with an increased number of blasts, and one had Chronic Myelomonocytic Leukemia. These five patients were different from the three patients who had a few ILC2 in the bone marrow, suggesting that the alterations in lineage are likely distinct among patients and as heterogeneous as the disease itself.

A significant difference was also found in ILC3 between healthy bone marrow and peripheral blood (p<0.001), with higher number of ILC3 in healthy bone marrow compared to peripheral blood (**Figure 4C**).

Additionally, ILC3 frequencies were performed by gender (**Supplementary Tables S6**, **S7**). Significant differences in ILC3 were observed between healthy bone marrow and peripheral blood for both female (p=0.013) and males (p=0.002) (**Figures 4D, E**). There were more ILC3 in the bone marrow compared to peripheral blood for both genders. No significant difference was found between genders for ILC3 in either bone marrow or peripheral blood (**Figures 4F, G**).

### Cytological analysis of ILC1, ILC2, and ILC3: comparison with T, B, and NK cells

Cytological analysis of ILC1 and ILC3 displayed small cells, with scarce cytoplasm, uniform nucleus, and condensed chromatin. As seen in **Supplementary Figures S5A, B**, these cells showed no evident differences between them through conventional morphology. However, ILC2 (**Supplementary Figure S5B**) exhibited an abundant cytoplasm with the presence of granules, a nucleus with condensed and irregular chromatin when compared to ILC1 (**Supplementary Figure S5A**) and ILC3 (**Supplementary Figure S5C**). Furthermore, except for ILC2, the cytological comparison carried out with the T, B and NK cells showed no morphological difference between these populations, thus making it not possible to differentiate them by conventional microscopy (**Supplementary Figures S5A-F**).

### Assessment of the bone marrow maturation curve for ILCs

**Figure 5:**
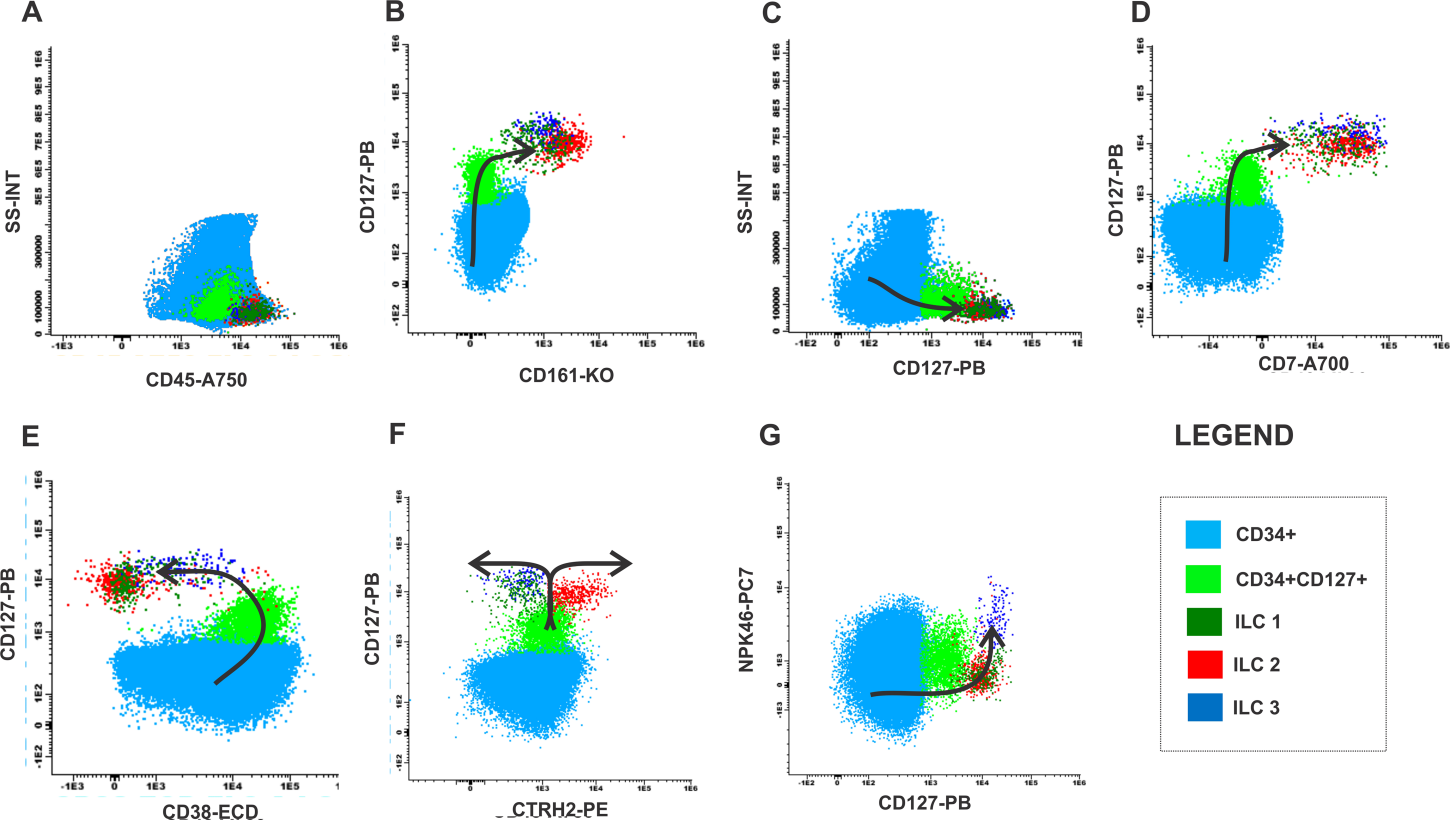
Maturation curve of ILCs from health bone marrow. **(A)** SS-INT x CD45-A750 graph identifying populations of CD34 myeloblasts (light blue), CD34 and CD127 immature lymphocytes (light green) and ILCs (dark green, red and dark blue), **(B)** CD127 PB x CD161-KO graph displaying the gain of CD127 and CD161 by the CD34 cells, **(C)** Graph SS-INT x CD127-PB displaying the gain of CD127 and CD7, **(E)** Graph CD127-PB x CD38-ECD displaying the downregulation of CD38 by the ILCs **(F)** Graph CD127-PB x CRTH2-PE displaying the gains the expression of CD127 and the ILC2 starts to express CRTH2 while ILC1 and ILC3 maintain the expression of CD127 **(G)** Graph CD127-PB x NKp46-PC7 displaying the gains the expression of CD127, the expression of NKp46 by ILC3 while ILC1 and ILC2 maintain CD127 expression while did not express NKp46-PC7.

We have established a possible maturation curve driven by mature ILCs from the CD34 precursor in healthy BM samples, according to **Figure 5** and **Supplementary Figure S2**. The population in light blue is the precursor cell (CD34, CD38, CD45) without lineage commitment and, the population in light green is the population committed to the lymphoid lineage (CD34, CD38, CD45, CD127). The dark green population demonstrates the ILC1 that expresses CD7, CD127, CD161 and loss CD38 expression (**Figures 5A–E**). The population in red represents ILC2, following the light green population (CD34, CD38, CD45, CD127) ILC2 starts to express CD7, CD127, CD161 with loss of CD38 expression (**Figures 5A–E**). Following the maturation arrow there is a gain in CRTH2 expression by ILC2 (**Figure 5F**) while the other subtypes remain negative for this marker. The dark blue population represents ILC3, following the light green population (CD34, CD38+CD45, CD127), ILC3 starts to express the markers CD7, CD127, CD161 with loss of CD38 expression (**Figures 5A–E**) and gain of NKp46 expression. Following the maturation arrow there is a gain in the expression of NKp46, a marker typically expressed by ILC3 (**Figure 5G**).

**Figure 6:**
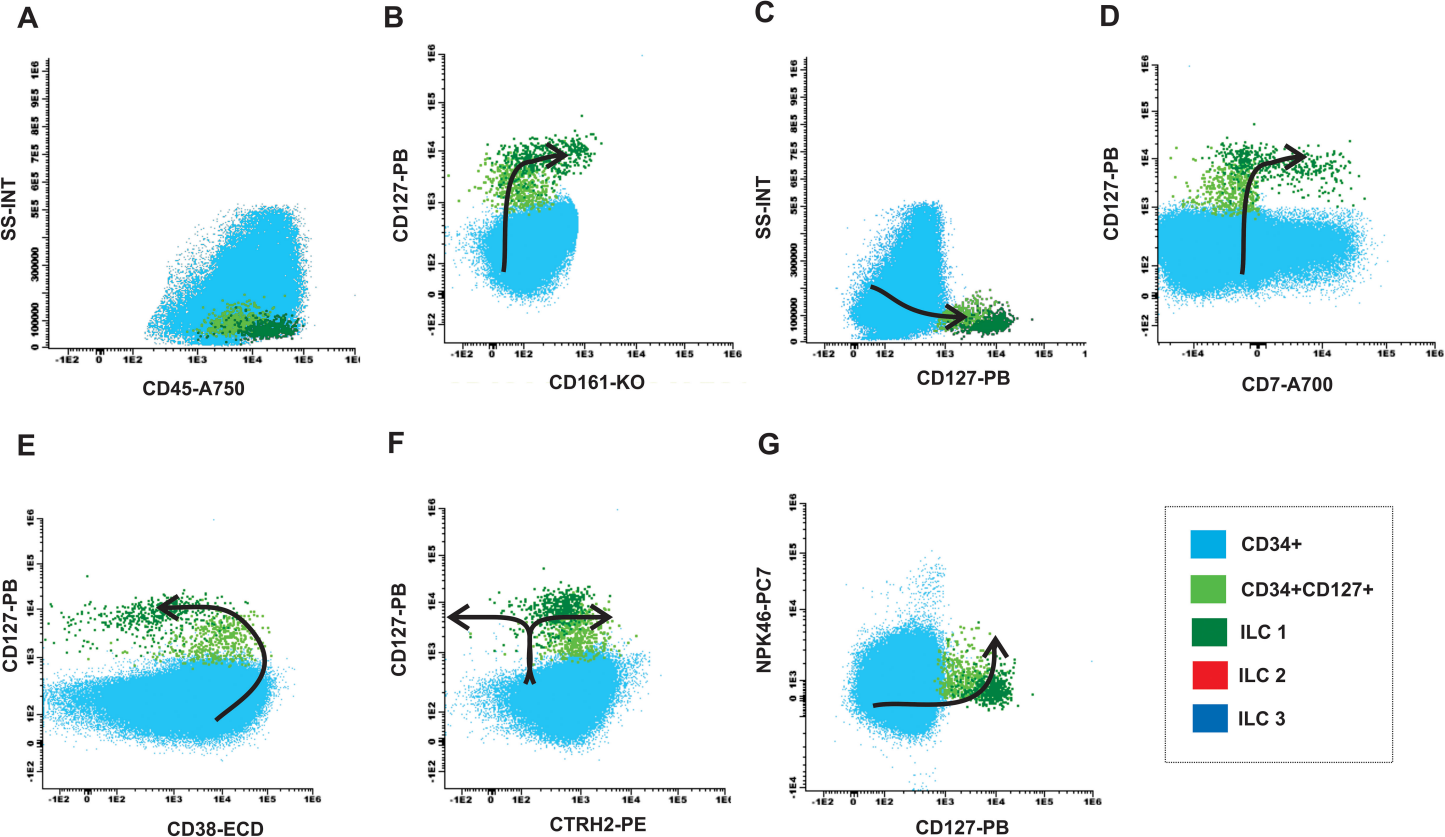
Maturation curve of ILCs from MDN bone marrow. **(A)** SS-INT x CD45-A750 graph identifying populations of CD34 myeloblasts (light blue), CD34 and CD127 immature lymphocytes (light green) and ILC1 (dark green), and absence of ILC2-ILC3 (red and dark blue), **(B)** CD127 PB x CD161-KO graph displaying the gain of CD127 and CD161 by the CD34 cells, **(C)** Graph SS-INT x CD127-PB displaying the gain of CD127 and CD7, **(E)** Graph CD127-PB x CD38-ECD displaying the downregulation of CD38 by the ILCs, **(F)** Graph CD127-PB x CRTH2-PE displaying the gains the expression of CD127 and the absence of ILC2 that express CRTH2 while ILC1 maintain the expression of CD127 **(G)** Graph CD127-PB x NKp46-PC7 displaying the gains the expression of CD127 and the absence of ILC3 that express NKp46 while ILC1 maintain CD127 expression.

In the MDN samples were identified an accumulation of myeloid progenitor cells (light blue population) CD34+CD45+CD127- (**Figures 6A–D**) and a decrease in the CD34+CD127+ population (**Figures 6A–D**). Furthermore, patients with MDN typically present heterogeneous expression, decreased or absent expression of CD38 in the CD34 progenitor population evidencing failure in the maturation curve as shown in **Figure 6E**. The curve is also incomplete due to the absence of the ILC2 and ILC3 populations (**Figures 6F, G**).

**Figure 7:**
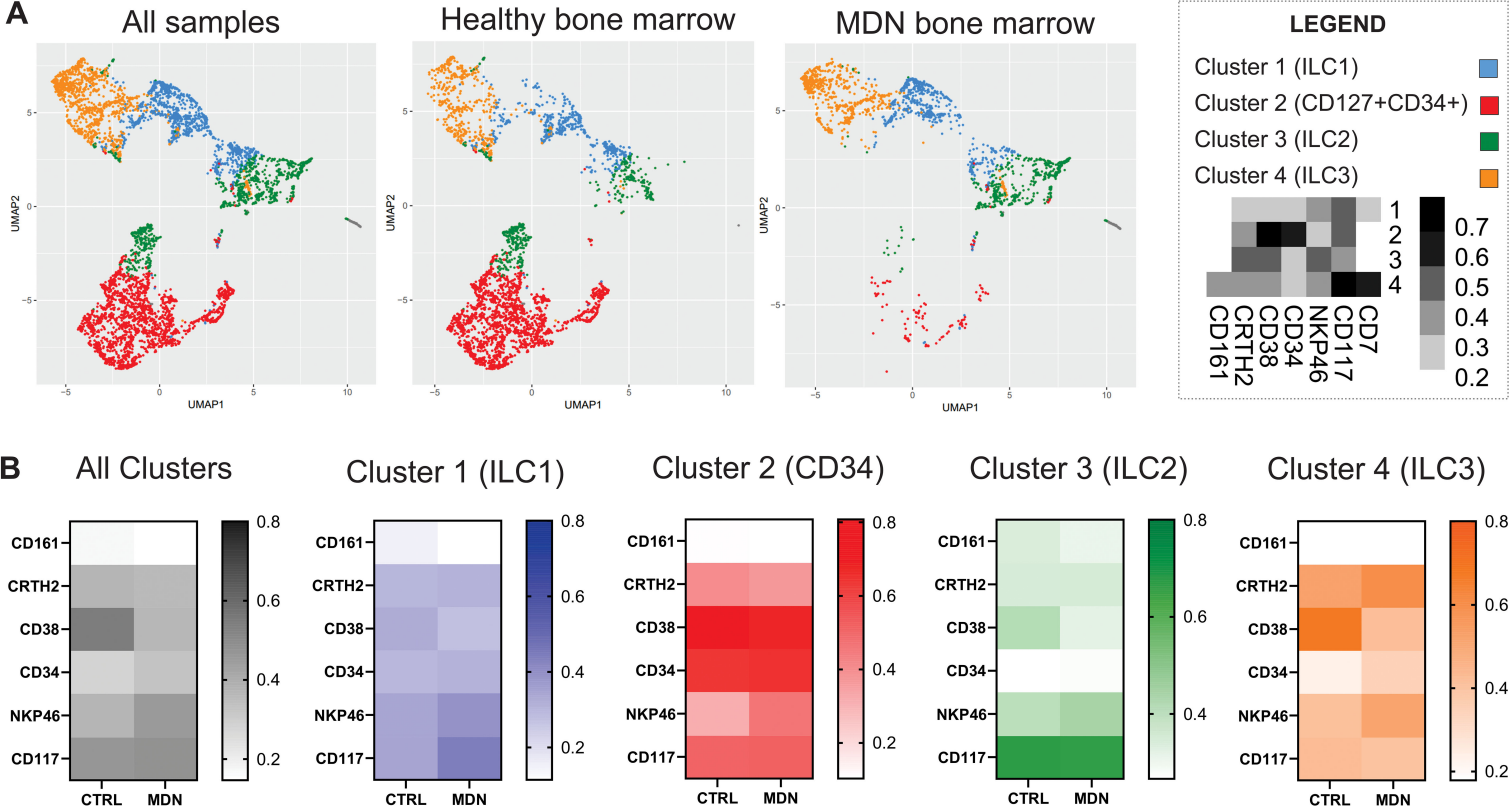
UMAP algorithms. **(A)** UMAP algorithms clusters for all samples, healthy bone marrow samples and MDN bone marrow, **(B)** Heatmaps for the expression of each maker analyzed for all cluster and individual clusters generated by the UMAP analysis.

Additionally, the populations were analyzed by the UMAP algorithms, and the results demonstrated a differential frequency between healthy and MDN bone marrow samples. The MDN bone marrow showed a significant loss of precursor population, which likely impacted the frequency of ILC2 and ILC3 (**Figure 7A**). Furthermore, the clusters identified by the UMAP, when compared to healthy bone marrow, not only showed a reduced frequency of cells but also differential expression of population markers. The most noticeable difference in all MDN clusters, was the downregulated frequency of CD38 and the upregulation of NKP46 (**Figure 7B**).

## Discussion

In the past decade, a family of lymphocytes known as ILCs (innate Lymphoid Cells) has been described (6, 7). However, studies on humans remain rare in the literature (23, 24). The scarcity of research is likely due to challenges in obtaining human bone marrow (BM) samples, and the few published studies have notable limitations. One of the primary challenges is accurately determining low-frequency cells in bone marrow samples, particularly for emerging and rare populations, such as innate lymphocytes.

This study identified, characterized, and presented percentage values for ILC1, ILC2 and ILC3 in bone marrow and peripheral blood from healthy individuals using flow cytometry. It also evaluated ILCs in MDN-BM, compared them to a healthy standard, and propose a maturation curve for these cells in the BM. Additionally, the morphology of innate lymphocytes in humans was demonstrated using conventional microscopy.

In this study, ILC1 were defined by the expression of CD45, CD7, CD127 and CD161 and the absence of LIN2, CTRH2 and NKp46. Our findings confirm that the same markers described in the literature for various other tissues are also expressed by ILC1 in the bone marrow and peripheral blood of healthy individuals (25–35). These data provide new insights, showing that ILC1 are present in both healthy PB and BM samples without significant differences between them or between genders. Additionally, we demonstrated that this population is present in the MDN-BM samples. In MDN samples, ILC1 did not exhibit any immunophenotypic difference or percentage differences compared to the healthy samples, even in individuals over 60 years of age, indicating that this population is not altered by the MDN.

ILC2 were defined by the expression of CD45, CD7, CD127, CD161, CTRH2 and the absence of LIN2, and NKp46. Our findings confirm that the same markers described in the literature for various other tissues are also expressed by ILC2 in bone marrow and peripheral blood of healthy individuals (1, 36–38). ILC2 is the only subtype of innate lymphocytes expressing CRTH2, a prostaglandin receptor associated with allergy and inflammation (34), highlighting its important role in inflammatory and allergic immune response (39, 40).

Few studies in the literature report the expression of ICOS and ICOS ligand on ILC2 (41). Additionally, it is known that lung dendritic cells express ICOSL, suggesting potential interactions between these cells (42, 43). However, this marker is only expressed when cells are exposed to specific stimuli. In our findings, we did not identify the expression of ICOS or ICOSL, likely due to the absence of cell stimulation. These results further suggest that these cells are not activated in the bone marrow.

The determination of ILC2 frequency adds valuable new information to the literature. This population was identified in all PB samples at lower frequency when compared to ILC1. Our findings corroborate data from Bonne-Anne et al., which reported a higher frequency of ILC1 in peripheral blood compared to the other ILC subsets (44). Mjösberg et al. also identified ILC2 in peripheral blood at low frequency and with the same phenotype observed in our study (34). This population was observed in healthy BM, even in individuals over 60 years old. However, we did not detect the ILC2 population in most bone marrow samples from MDN patients.

ILC2 is related with inflammatory and allergic responses, which is linked to their expression of CRTH2, a prostaglandin receptor (36, 40, 45–47). Prostaglandins are involved in several critical processes, including immunological responses, regulation of chemotactic activity, and metastatic progression (48, 49). The absence of this population in most MDN-BM may suggest a partial failure in innate immune processes and could indicate a compromised response to inflammatory and infectious processes, which correlate clinically with high rates of bacterial infection in these patients (50, 51). Furthermore, ILC2 secrete IL-13, which plays a crucial role in recruiting regulatory cells myeloid-derived and type 2 macrophages (M2). These cells are important for maintaining controlled inflammatory levels in the BM microenvironment and may influence the differentiation of dysplastic cells (52, 53).

The release of IL-13 plays a crucial role in the production of TGF-β by myeloid lineage-derived suppressor cells. Along with Th2 cytokines, this interaction activates M2 macrophages through an alternative pathway. Additionally, IL-13 is pivotal in orchestrating the Th2 immune response, and in the differentiation of B cells (53, 54). The observed reduction of ILC2 in MDN patients may disrupt the availability of IL-13, thereby contributing to impaired immune process cascades.

In mice ILC2-derived IL-13 promotes a rapid immune response and enhances bacterial clearance following infection with *S. pneumoniae* (55). Furthermore, a study by Chun et al. demonstrated an increase in ILC2 in the small intestine and peritoneal cavity, accompanied by a decrease in the liver after septic shock (56). This sepsis mouse model also showed increased presence of ILC2 in the lungs, where they secrete IL-9. This secretion of IL-9 protects lung epithelial cells by preventing the activation of inflammatory processes (57).

Additionally, ILC2 is the predominant ILC subtype in the skin, which frequently serves as the entry for bacteria related to severe infections (58, 59). *S. aureus* infections can activate ILC2 and promote a Th2 immune response (57). These findings highlight the complex contribution of ILC2 in bacterial infections, with potential organ-specific effects.

ILC3 are characterized by the expression of CD7, CD45, CD127, CD161 and NKp46 and the absence of LIN2 and CRTH2. These cells are present in healthy BM and PB, even in individuals over 60 years old.

Similarly to ILC2, ILC3 was also not identified in most MDN-BM samples. This population is related to tissue homeostasis and plays significant role in immunity against extracellular bacteria and fungi. Alterations in this population have been reported in chronic inflammation of the skin, lungs, and intestine (60–65).

ILC3 is responsible for the secretion of IL-17, which is essential for defending the organism against extracellular bacteria and fungi by recruiting neutrophils to infected areas (24, 66–68). The reduction or absence of this population can decrease IL-17 production, impairing the recruitment of neutrophils to infection sites. This observation aligns with clinical symptoms of MDN patients, 40% of whom experience high rates of recurrent bacterial infections (50, 69).

ILC3 also secret IL-22, which induces the expression of pro-inflammatory molecules, such as IL-1, IL-6, IL-8, IL-11 and GM-CSF (35). Studies have shown that IL-22-deficient mice exhibit exacerbated intestinal inflammation and epithelial barrier damage leading to generalized infection (70, 71). In addition, IL-22, deficiency affects the production of IL-17 by ILC3, which may contribute to increased *Candida albicans* fungal infections in experimental mouse models (72). In *Streptococcus pneumoniae* infections, ILC3 accumulate in the lungs, providing IL-22 and aiding in infection resolution (73).

Thus, the decrease or absence of ILC3 in MDN-BM patients may contribute to the recurrent bacterial and opportunistic infections observed in these individuals. However, to determine whether ILCs are involved in hematopoietic dysplasia, it is essential interesting to understand the maturation pathways of these cells from their most immature progenitor.

We provide insights into the maturation path of ILC, starting from the progenitor cells expressing CD34 and CD38 to more mature precursor expressing CD127 and CD161, in the absence Lineage 2 markers, a characteristic common to all ILC subtypes. In addition, we illustrated the maturation path of ILC2 as they acquire CRTH2 expression. It is possible that ILC2 shifts upon gaining CRTH2, while the other two subtypes, ILC1 and ILC3 shift in the opposite direction.

The path taken by ILC3 in acquiring NKp46 expression can also be visualized in our strategy. Additionally, we compared this pattern with MDN-BM patients. Our data indicate that the absence of ILC2 and ILC3 may represent a failure in the maturation curve of these patients compared to healthy individuals. Furthermore, we observed the accumulation of the immature CD34 positive, but CD127 negative myeloid population and the decrease in the CD34 and CD127-positive population in MDN-BM patients. This characterizes lack of heterogeneity and a failure in the conventional maturation pattern when compared to the healthy BM.

Myelodysplastic neoplasia (MDN) comprises a heterogeneous group of clonal disorders characterized by progressive damage to the bone marrow microenvironment, leading to impaired cellular differentiation across one or more hematopoietic lineages (74–77). In MDN, hematopoietic stem and progenitor cells are disrupted in the presence of dysplastic cells and functional abnormalities in hematopoietic stem and progenitor cells (HSPCs). This dysfunction compromises the bone marrow’s capacity to generate various immune cell populations, which may include innate lymphoid cells (ILCs). The progenitor cells responsible for ILC differentiation may be directly inhibited or outcompeted by dysplastic clones, or their development may be impaired by the altered microenvironment.

A hallmark of MDN is the presence of a chronic inflammatory state within the bone marrow, often accompanied by an imbalanced cytokine milieu. Elevated levels of pro-inflammatory cytokines, such as TNF-α, IL-6, and IFN-γ, along with altered growth factors, may have immunosuppressive or cytotoxic effects on ILC precursors. This dysregulated inflammatory environment can impair both the differentiation and survival of ILCs, potentially contributing to their reduced numbers or absence in MDN bone marrow.

Furthermore, genetic and epigenetic alterations commonly observed in MDN may disrupt immune homeostasis and affect ILC development. Mutations in key regulatory genes or aberrant epigenetic modifications may impair the lineage commitment and function of progenitor cells, further contributing to the depletion of ILC subsets, even though no specific mutations on ILC precursor have been described until now. These mechanisms may explain the observed absence of ILC2 and ILC3 in the bone marrow of most MDN patients. Notably, ILC2 and ILC3 populations are present in the bone marrow of healthy individuals over 60 years old, suggesting that their depletion in MDN is disease-related rather than age-dependent.

The analysis using UMAP algorithms revealed a loss in the precursor population defined by us (LIN2-CD45+CD34+CD38+CD127+) and a reduced expression of CD38 in all clusters analyzed. CD38 is a multifunctional enzyme and receptor involved in the regulation of intracellular calcium signaling, which is crucial for cell activation and proliferation. Reduced CD38 expression could impair these signaling pathways, potentially affecting the activation and development of precursor cells (77). Additionally, reduced expression of CD38 in precursor cells could lead to impaired immune cell development and a weakened immune response. CD38 is important for the regulation of hematopoiesis, and its downregulation in bone marrow precursor cells could disrupt this process, potentially leading to deficiencies in certain blood cell lineages and contributing to hematological disorders such as MDN (78).

Furthermore, the UMAP algorithm revealed significant upregulation of NKP46 in MDN-BM-ILCs, which could be associated with increased production of inflammatory cytokines. While this might be beneficial in fighting infections, it could also contribute to chronic inflammation, which is characteristic of MDN (79).

Other studies show that high-risk MDN patients with a worse prognosis display a deficiency in NK cells. These cells, which are part of the innate immune system and derived from common lymphoid progenitor, indicate that MDN dysplasia can compromise the lymphoid compartment.

Our study reports and suggests a possible ILC maturation curve using 40 BM samples from healthy individuals, strengthening our data. We have identified morphological differences in ILC2 allowing their identification through conventional microscopy. ILC2 show abundant cytoplasm with granules, while other lymphocytes exhibit a lymphoid morphology that is indistinguishable by light microscopy. Similar to eosinophils and basophils, which also contain abundant granules, ILC2 are also involved in allergic processes and parasitic infections. The granules in ILC2 cytoplasm may be inflammatory mediators involved in these processes.

In conclusion, determining reference values for innate lymphocytes, as well as their characterization and maturation profiles in healthy BM, provides new insights into how the immune response and tissue homeostasis are regulated by these cells. Furthermore, understanding the typical pattern allows for the creation of comparative profiles with various other pathologies other than MDN, as already demonstrated by the role of innate lymphocytes in patients with Acute Myeloid Leukemia and cancer (80, 81).

## Data Availability

The raw data supporting the conclusions of this article will be made available by the authors, without undue reservation.
