# Supplementary material for: Innate lymphoid cells in bone marrow and peripheral blood of healthy individuals and in bone marrow of patients with myelodysplastic syndromes
Source: Front Immunol. 2025 Jun 11;16:1568875. doi: 10.3389/fimmu.2025.1568875 (PMC12187685; doi:10.3389/fimmu.2025.1568875)
Supplement: Supplementary file 1 [file DataSheet1.docx]

**SUPPLEMENTARY FIGURES**


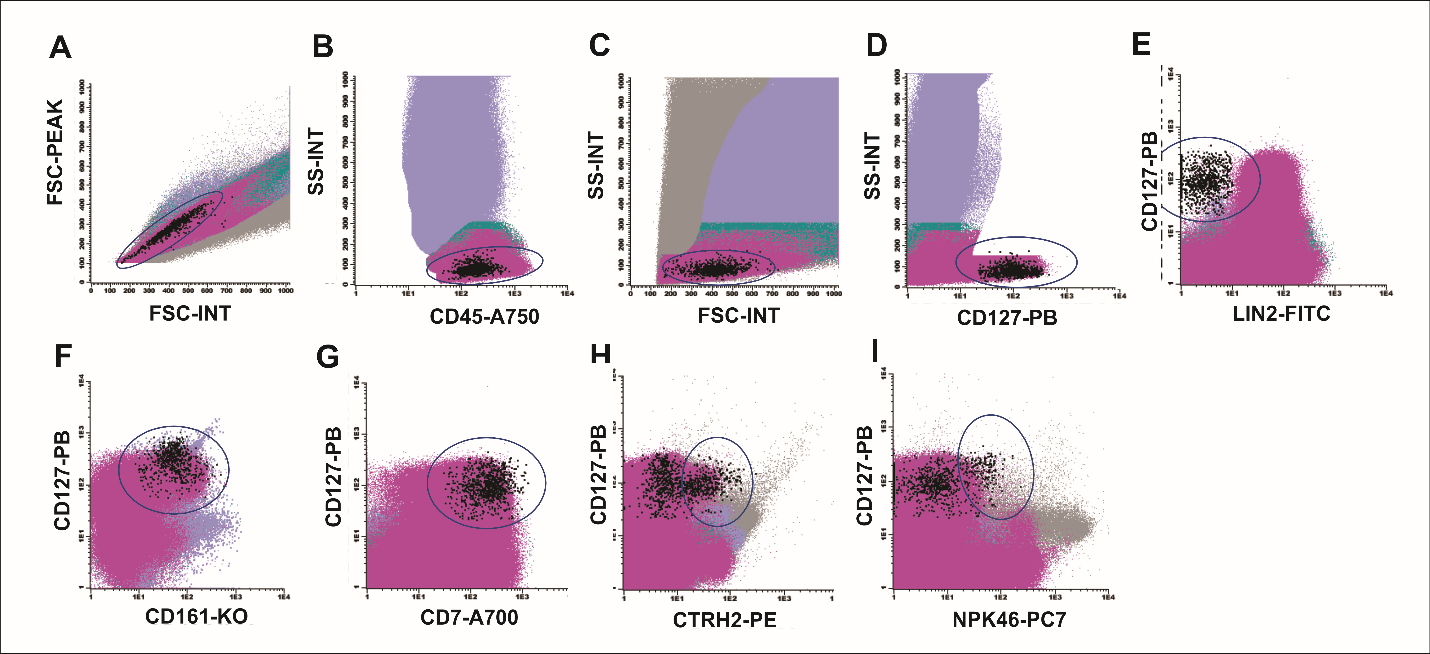


**Fig S1. Analysis strategy used to identify ILCs.**

**(A)** FS-PEAK X FS-INT gate was used to remove the doublets population, (**B)** SS-INT X CD45-A750 gate selected the population of lymphocytes with strong expression of CD45-A750 and low complexity, (**C)** SS-INT X FS-INT gate selected the viable cells, (**D)** SS-INT X CD127-PB gate select the lymphocyte population**, (E)** CD127-PB X LIN2-FITC gate select the population CD127 positive and LIN2 negative, **(F)** CD127-PB X CD161-KO gate selected the double positive population for these markers (**G)** CD127-PB X CD7-A700 gate selected the population double positive for these markers**. This population was defined as ILCs.**

**(H)** CD127-PB and CRTH2-PE gate selected the double positive population for these markers and was identified **as ILC2**, **(I)** CD127-PB X NKp46-PC7 gate selected the population double positive for these markers and was identified as **ILC3.** The population negative for CRTH2-PE and NKp46-PC7 was identified as **ILC1.**


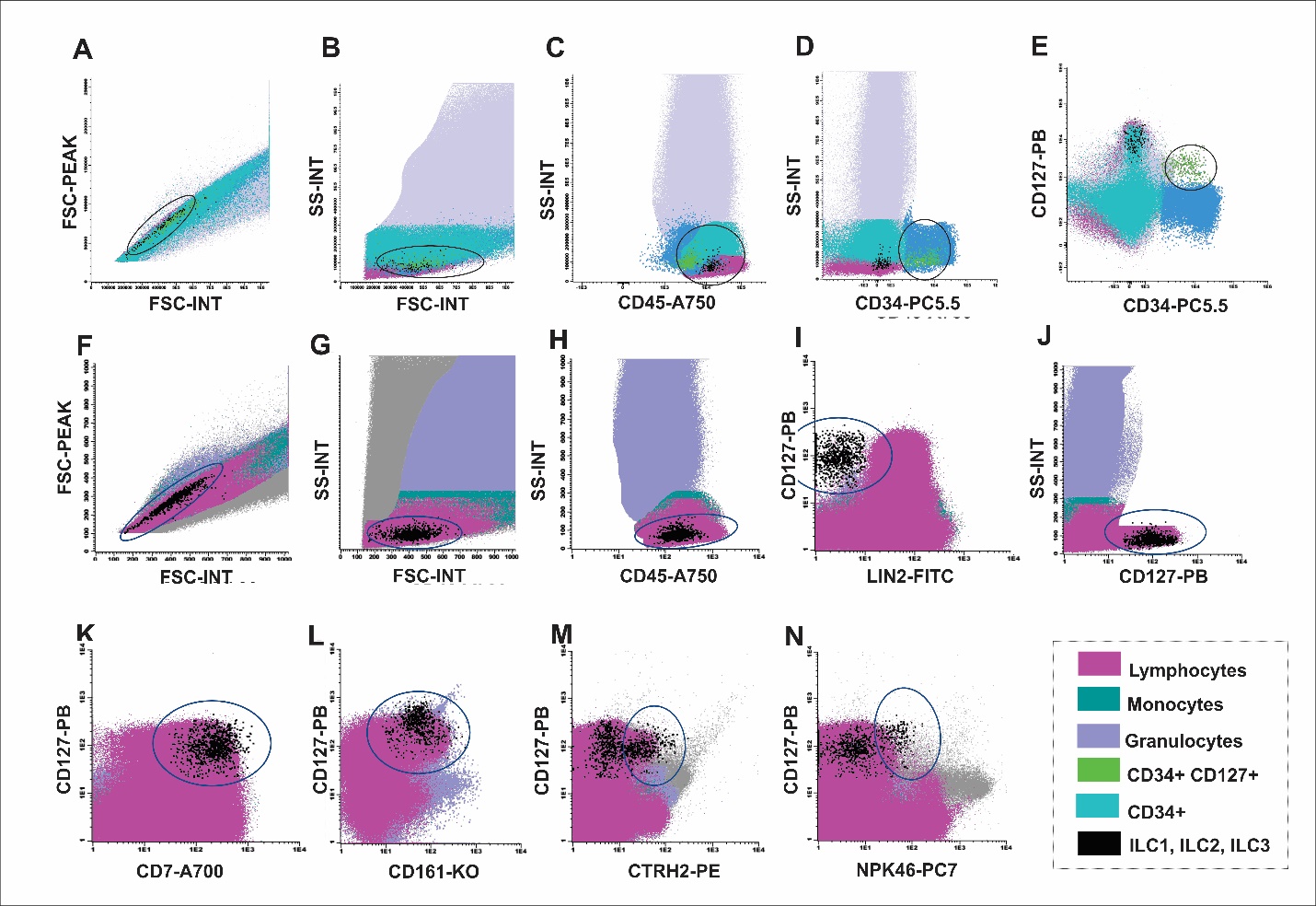


**Fig S2. Strategy used to construct the ILCs maturation curve.**

**(A)** Graph FS-Peak x FS-INT used to remove the doublet population, **(B**) Graph SS-INT x FS-INT used to select viable cells, **(C)** Graph CD45-A750 x SS-INT used to select leukocytes, **(D)** Graph SS-INT x CD34-PC5.5 used to select myeloblasts and lymphoblasts, **(E)** Graph CD127-PB x CD34-PC5.5 used to separate te doble positive population,**(F)** Graph FSC-Peak x FSC-INT used to remove the doublet population**, (G)** Graph SS-INT x FS-INT used to select viable cells, **(H)** Graph CD127-PB x LIN2-FITC used to exclude lineage cells, **(I)** Graph SS-INT x CD127-PB used to select lymphoid population **(J)** Graph CD7-A700 x CD127-PB used to select the double positive population, **(K)** Graph CD161-KO x CD127-PB used to select ILC, **(L)** Graph CTRH2-PE x CD127-PB used to separate ILC2 from ILC1 and ILC3 **(M)** Graph NPK46-PE x CD127-PB used to separate ILC3 from ILC1 and ILC2**.**


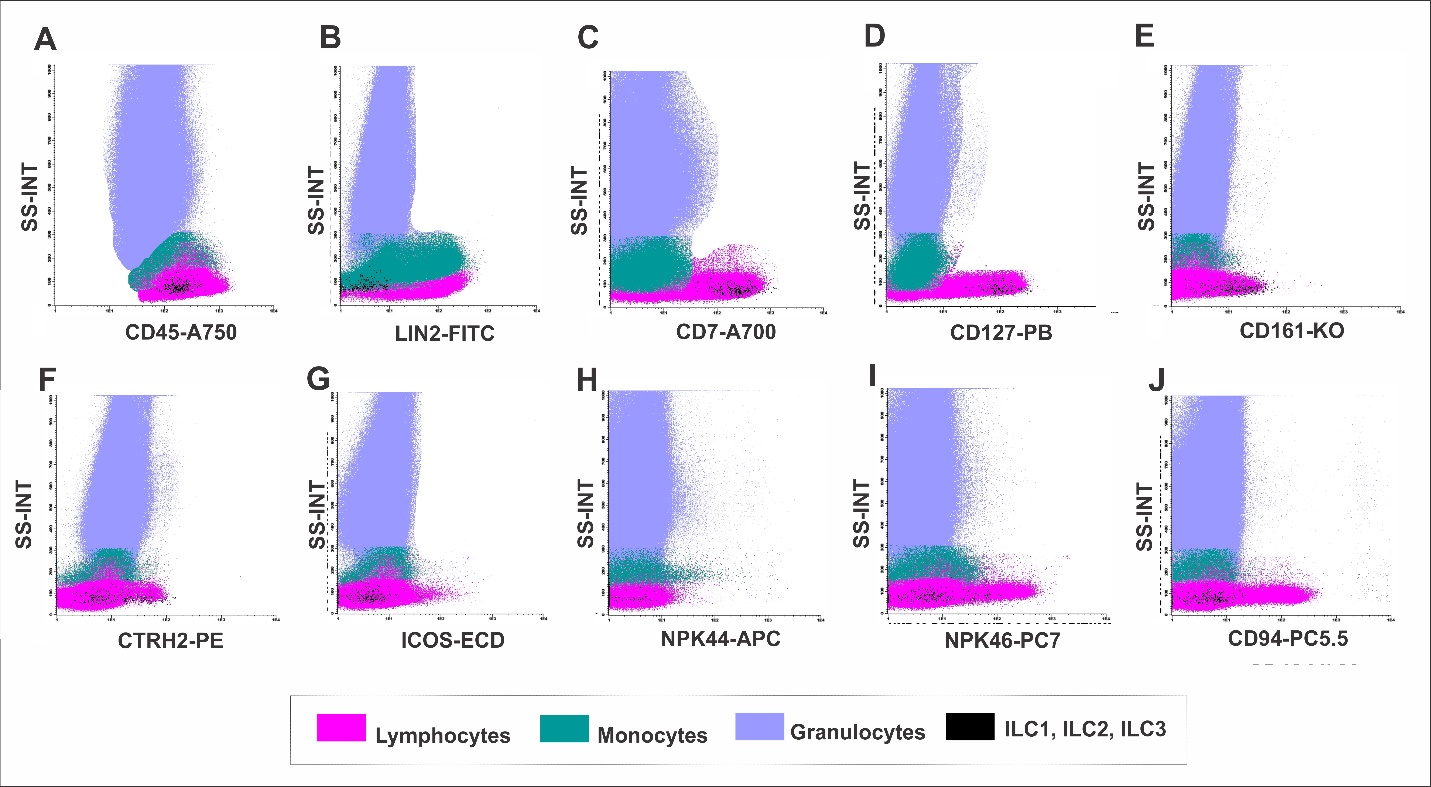


**Fig S3. Strategy showing the use of populations for internal control.**

**(A)** Graph SS-INT vs CD45-A750, the CD45 is known to be expressed for all leukocytes, **(B)** Graph SS-INT vs LIN2-FITC, LIN2 is known to be positive for lymphocyte and monocyte populations and absent in ILC1, ILC2, ILC3 and granulocytes, **(C)** Graph SS-INT vs CD7 A700, CD7 is known to be expressed by T cells and ILCs types 1, 2 and 3 and absent in granulocytes, monocytes and B cells,**(D)** Graph SS-INT vs CD127-PB, CD127 known to be expressed by lymphocytes and ILC1, ILC2 and ILC3 and absent in granulocytes and monocytes, **(E)** Graph SS-INT vs CD161-KO, CD161 is known to be expressed by memory and effector T cells, γδ T cells, NK cells and ILC1, ILC2, ILC3 and absent in granulocytes, monocytes and part of the lymphocytes,**(F)** Graph SS-INT vs CTRH2-PE, CTRH2 is known to be expressed by cytotoxic T cells and ILC2 and absent in monocytes, granulocytes and ILC1 and ILC3, ,**(G)** Graph SS-INT vs ICOS-ECD, ICOS is known to be expressed in activated T cells, a subset of thymocyte and, negative for the other populations, **(H)** Graph SS-INT vs NKP44-APC, NPK44 is known to be present in activated NK cells, a subpopulation of ILC3, which we were not able to identify in human peripheral blood, and absent in granulocytes, monocytes, lymphocytes and ILC1, ILC2, **(I)** Graph SS-INT vs NKP46-PC7, NPK44 is known to be expressed by lymphocytes and ILC3 and absent in granulocytes, monocytes and ILC1 and ILC2, **(J)** Graph SS-INT vs CD94-PC5.5, CD94 is known absent in granulocytes, monocytes, ILC1, ILC2, ILC3 and expressed by NK cells and a subset of γδ T cells.


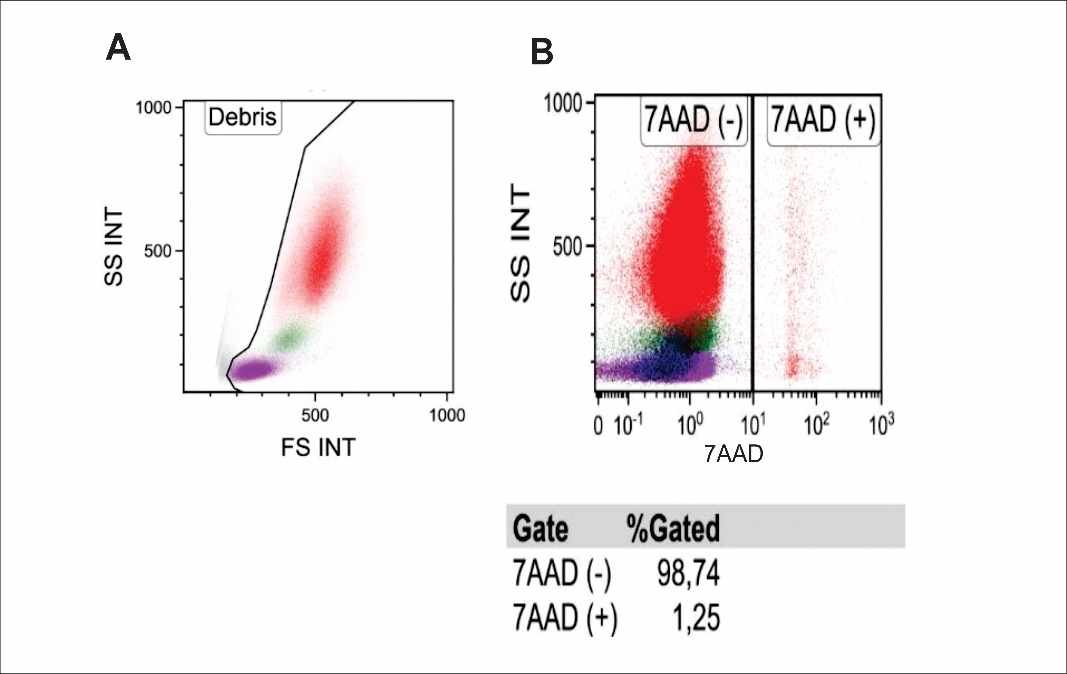


**Fig S4. Strategy showing the quantification of viable cells. (A)** Total cell region (total cells), displaying the exclusion of debris by the gate in SS INT X FS INT, **(B)** In the region of total cells (without debris), two populations were classified according to the expression of the 7AAD dye, 7AAD (+) non-viable cells and 7AAD (-) viable cells.


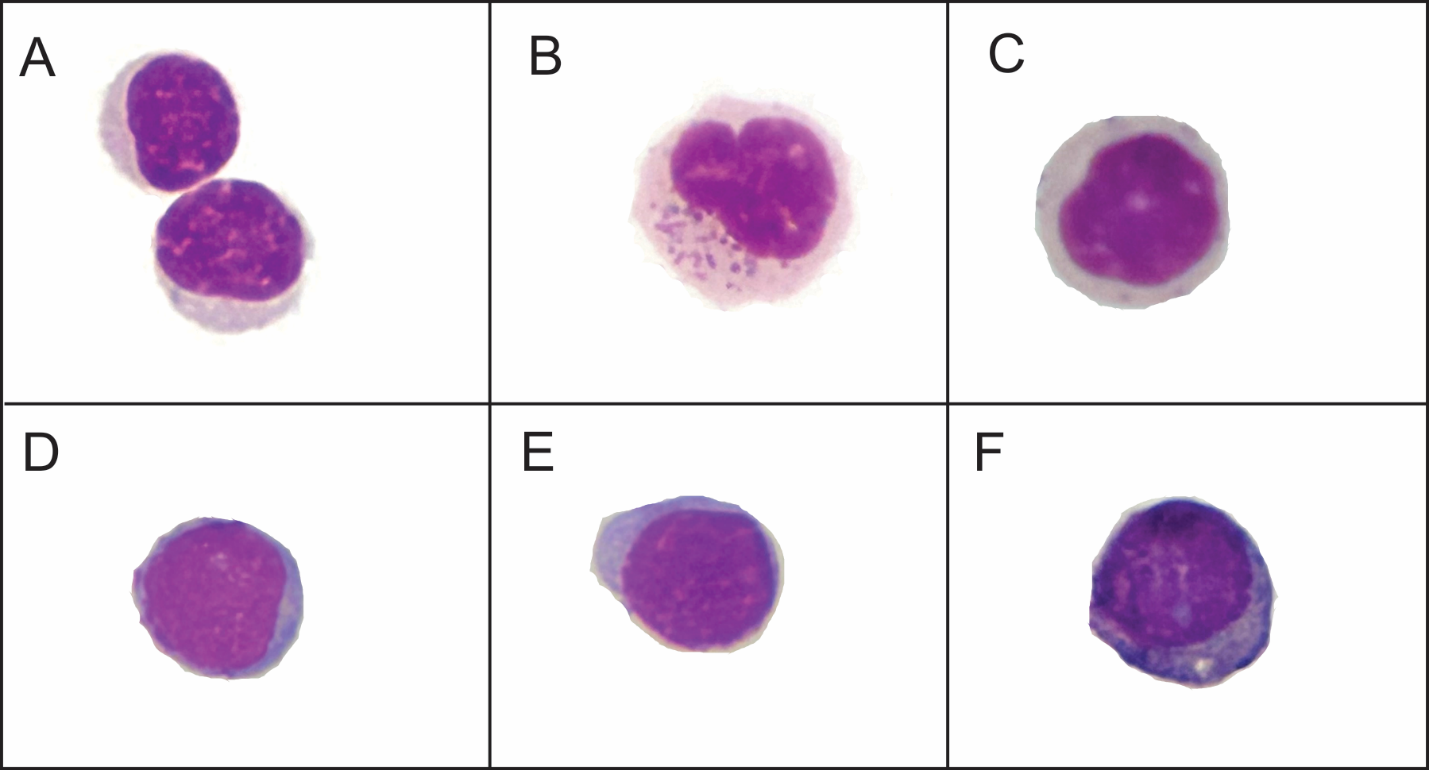


**Fig S5. Morphological analysis of ILCs (A)** Morphological analysis of ILC1, **(B)** Morphological analysis of ILC2, **(C)** Morphological analysis of ILC3, **(D**) Morphological analysis of T cells, **(E)** Morphological analysis of B cells, **(F)** Morphological analysis of NK cells. Rosenfeld staining (100x magnification). To generate this image, we have used a mask to remove the background and highlight the cell.
