# Supplementary material for: Innate lymphoid cells in bone marrow and peripheral blood of healthy individuals and in bone marrow of patients with myelodysplastic syndromes
Source: Front Immunol. 2025 Jun 11;16:1568875. doi: 10.3389/fimmu.2025.1568875 (PMC12187685; doi:10.3389/fimmu.2025.1568875)
Supplement: Supplementary file 2 [file DataSheet2.docx]

**Supplementary Tables**

**Table S1 –** Demographic data on NMD patients and disease classification

**Table S2 -** Result of differences between samples of healthy bone marrow and peripheral blood in ILC1 in samples from the female population.

| **GROUP** | **ILC1 (Female)** | |  |
| --- | --- | --- | --- |
|  | **HEALTHY  BONE MARROW** | **PERIPHERAL BLOOD** | p |
|  | **Median** | **Median** |  |
| **ILC1 (LR) %** | 0.042 (0.011 – 0.074) | 0.0405 (0 – 0.1) | 0.988 |
| **ILC1 (EVN)** | 247  (94 -735) | 577  (0 - 1644) |  |

**LEGEND: LR=Lymphocyte region; EVN= Events Number**

**Table S3 -** Result of differences between samples of healthy bone marrow and peripheral blood in ILC1 in samples from the male population.

| **GROUP** | **ILC1 (Male)** | |  |
| --- | --- | --- | --- |
|  | **HEALTHY  BONE MARROW** | **PERIPHERAL BLOOD** | p |
|  | **Median** | **Median** |  |
| **ILC1 (LR) %** | 0.034  (0.011 – 0.49) | 0.04  (0.007 – 0.1) | 0.919 |
| **ILC1 (EVN)** | 0,034  (0,011 - 0,49) | 0,04  (0,007 - 0,1) |  |

**LEGEND: LR=Lymphocyte region; EVN= Events Number**

**Table S4 -** Result of differences between samples of healthy bone marrow and peripheral blood in ILC2 in samples from the female population.

| **GROUP** | **ILC2**  **(Female)** | | | |
| --- | --- | --- | --- | --- |
|  | **HEALTHY  BONE MARROW** | | **PERIPHERAL BLOOD** | p |
|  | **Median** | **Median** | |  |
| **ILC2 (LR) %** | 0.0165 (0 – 0.065) | 0.01 (0 – 0.085) | | 0.405 |
| **ILC2 (EVN)** | 115  (0-657) | 174,5  (0-682) | |  |

**LEGEND: LR=Lymphocyte region; EVN= Events Number**

**Table S5 -** Result of differences between samples of healthy bone marrow and peripheral blood in ILC2 in samples from the male population.

| **GROUP** | **ILC2**  **(Male)** | | |
| --- | --- | --- | --- |
|  | **HEALTHY  BONE MARROW** | **PERIPHERAL BLOOD** | p |
|  | **Median** | **Median** |  |
| **ILC2 (LR) %** | 0.018  (0.003 - 0,041) | 0.016  (0 – 0.085) | 0.183 |
| **ILC2 (EVN)** | 119  (30 - 535) | 105  (0-457) |  |

**LEGEND: LR=Lymphocyte region; EVN= Events Number**

**Table S6-** Result of differences between samples of healthy bone marrow and peripheral blood in ILC3 in samples from the female population.

| **GROUP** | **ILC3**  **(Female)** | | |
| --- | --- | --- | --- |
|  | **HEALTHY  BONE MARROW** | **PERIPHERAL BLOOD** | p |
|  | **Median** | **Median** |  |
| **ILC3 (LR) %** | 0.022 (0.05 – 0.035) | 0.01 (0 – 0.06) | **0.013** |
| **ILC3 (EVN)** | 171  (34-296) | 174  (0-1101) |  |

**LEGEND: LR=Lymphocyte region; EVN= Events Number**

**Table S7-** Result of differences between samples of healthy bone marrow and peripheral blood in ILC3 in samples from the male population

| **GROUP** | **ILC3**  **(Male)** | | |
| --- | --- | --- | --- |
|  | **HEALTHY  BONE MARROW** | **PERIPHERAL BLOOD** | p |
|  | **Median** | **Median** |  |
| **ILC3 (LR) %** | 0.023  (0.0015 – 0.1) | 0.01  (0.003 – 0.03) | **0,002** |
| **ILC3 (EVN)** | 190  (50 - 716) | 72  (38-304) |  |

**LEGEND: LR=Lymphocyte region; EVN= Events Number**
